# Editorial: MYC as a disease target beyond cancer

**DOI:** 10.3389/fcell.2024.1467372

**Published:** 2024-08-22

**Authors:** Jonathan R. Whitfield

**Affiliations:** Models of Cancer Therapies Laboratory, Vall d'Hebron Institute of Oncology (VHIO), Vall d’Hebron Barcelona Hospital Campus, Barcelona, Spain

**Keywords:** MYC, targeting, transcription factor, non-oncologic diseases, therapy

MYC is a highly pleiotropic transcription factor involved in multiple cellular and developmental processes, but it is most renowned for its role in driving tumorigenesis. Hence, it is currently subject to intense efforts towards its targeting for the treatment of cancer, and while it was long considered undruggable, there are now 3 direct inhibitors in clinical trials, one of which recently successfully passed Phase I. There are many existing reviews on its role in cancer and strategies for its targeting, therefore here we focus on all the other diseases and conditions to which its inhibition–or activation or overexpression in some cases–could be applied.

There are a significant number of publications linking MYC to a wide variety of diseases, some with only preliminary data showing modulated MYC expression in disease models or patient samples, while others describe inhibition, knock-down or overexpression of MYC to demonstrate a key role in disease development or progression. As MYC inhibitors advance in their clinical testing, one can hope that they will soon be applied beyond cancer patients. It is curious that some of the earliest trials were indeed in a non-oncological setting, using MYC antisense for the treatment of heart restenosis.

With this intense focus on MYC inhibition in cancer, it is easy to lose track of all the other diseases in which MYC is key. The objective of this Research Topic was to bring together reviews and original research papers that link non-oncological pathologies with MYC.

This Research Topic kicks off with a review of the literature linking MYC to various diseases and conditions beyond cancer (Zacarías-Fluck et al.). This is an overview of MYC and its multiple physiological functions, which are also described in more detail in an additional review in this Research Topic by Kumar Jha et al., that extensively discusses the MYC amplifier model. Our introductory review (Zacarías-Fluck et al.) then describes how MYC often becomes a central hub used by oncogenic drivers to modulate many cellular processes, and that these same multiple and pleiotropic functions of MYC implicate it in the aetiology of a wide variety of diseases. These range from conditions of ‘normal development gone wrong’, as in the case of bone development disorders, to those where mutations in upstream signalling pathways drive de-regulated MYC expression or activation and the development of a pathological condition.

Then, the Research Topic dives into specific conditions, with reviews on MYC’s role in regeneration, aging, mitochondrial diseases, obesity, and endometriosis. Indeed, MYC has a well-known role in regeneration across the animal kingdom, and this is discussed by Ascanelli et al. along with the potential to activate MYC in non-regenerative tissues for therapeutic purposes. This is extended to *Drosophila* in a review by Serras and Bellosta, who focus on the regenerative process in flies, and the utility of this model for understanding human tissue repair (Serras and Bellosta).

MYC in aging is a rather more controversial topic with seemingly contrasting data between MYC haploinsufficient and MYC KO models, mentioned in the review by Zacarías-Fluck et al., and with the MYC KO data discussed at length in an additional review by Prochownik and Wang.

MYC also plays a role in stem cell renewal, and the MYC-SIRT1 axis is described by Fan and Li to have a part to play both in cancer and normal embryogenesis, the latter suggesting that MYC could be a therapeutic target in developmental diseases.

In addition, a perspective piece by Nothnik et al. discusses MYC’s role in endometriosis, a disease that affects many women, causing pain and reduced quality of life, and presents some preliminary data showing that MYC inhibition reduces endometriotic cell proliferation and viability *in vitro*.

Another article of the Research Topic sheds light on the relatively unexplored role of MYC in mitochondrial diseases. In fact, while the association of MYC upregulation with mitochondrial dysfunction is quite clear, Purhonen et al. review 2 decades of literature and the role of MYC in various mitochondrial diseases, identifying key questions that are still unanswered.

Two articles in the Research Topic discuss obesity. Nevzorova and Cubero refer to “moonlighting” MYC due to its many jobs within a cell, and describe mechanisms for the development of obesity and the implication of MYC in them. Given the rapidly increasing incidence of obesity–and the subsequent impact on health and healthcare systems–it is provocative to think that MYC inhibitors could have a place in its treatment. However, an original research paper included in this Research Topic suggests that inhibition of MYC is associated with weight gain. In this article, knockout of MYC in mouse endothelial cells leads to progressive increase in body weight during aging, while overexpression of MYC attenuates diet-induced obesity (Machi et al.). On the other hand, oral administration of the small molecule MYC inhibitor 10058-F4 to obese mice was previously shown to reduce obesity (Luo et al., 2021). It appears that further studies with additional obesity models and MYC inhibitors are still needed to clarify this topic.

Additional original research papers in the Research Topic also provide new data pointing to a role for MYC in polycystic kidney disease and neonatal lung disease, as well as to its involvement in the process of self-renewal, and the control of nucleolar function and the somatotropic axis. The study by Harafuji et al. for example, relates to autosomal recessive polycystic kidney disease, a severe hepato-renal disorder that causes childhood morbidity. The authors show that MYC is overexpressed in kidneys from disease patients and find an association between MYC expression levels and renal cyst development in mouse models. The next step will be to show that MYC inhibitors can modulate disease progression.

Intra-amniotic inflammation is associated with morbidity at an even earlier age, causing pre-term births and chronic lung disease of prematurity. In a research article, Tan et al. use the MYC inhibitor 10058-F4 to treat a model of intra-amniotic inflammation in pregnant rats caused by LPS. Here, MYC expression is associated with the intra-amniotic inflammation in neonatal tissues, and treatment with the MYC inhibitor ameliorates many of the effects of LPS.

Furthermore, roles of MYC in additional processes may hint at new disease applications, for example, its control of nucleolar function could be linked to cancer and ribosomopathies (Manara et al.), while a role in self-renewal of oesophogeal epithelium basal cells (Hishida et al.) could link to regeneration after injury or for repair, as also mentioned above.

Finally, a role in the regulation of the somatotropic axis through miRNA-mediated IGF1 downregulation suggests that the link between MYC, aging and several diseases (such as cancer, cardiovascular disease, diabetes, osteoporosis, and neurodegeneration) is a convoluted one and requires further investigation to more clearly define it (Petrashen et al.).

Overall, it is an exciting and optimistic time for cancer researchers in the MYC inhibitor field, and hopefully clinical success there will soon lead to the application of MYC inhibitors in multiple and diverse diseases. There has so far been far less focus on MYC overexpression or activation for disease modulation, but as described in the Research Topic, this may be therapeutic in conditions requiring tissue regeneration, and as such may be a new challenge for the MYC field. We are eagerly looking forward to seeing all the research into MYC making a difference for as many diseases as possible.
